# The Mediating Role of Hesitancy in the Associations Between Mental Disorders and Social Support Seeking During the COVID-19 Pandemic

**DOI:** 10.3390/bs14110979

**Published:** 2024-10-22

**Authors:** Qinghua Yang, Muniba Saleem, Elizabeth Dobson, Suzanne Grimmesey

**Affiliations:** 1 Department of Communication Studies, Bob Schieffer College of Communication, Texas Christian University, Fort Worth, TX 76129, USA; 2 Department of Communication, University of California, Santa Barbara, CA 93106, USA; msaleem@comm.ucsb.edu; 3 Communication Department, School of Social and Behavioral Sciences, Casper College, Casper, WY 82601, USA; elizabeth.dobson@caspercollege.edu; 4 Santa Barbara County Department of Behavioral Wellness, Santa Barbara, CA 93110, USA; suzkirk@co.santa-barbara.ca.us

**Keywords:** pandemic, mental health, social support, hesitancy, channel complementarity, community

## Abstract

The COVID-19 pandemic has consequential impacts on not only physical but also mental health. However, the types of social support that individuals with mental health needs sought during the pandemic and their underlying reasons for it are not well known. Drawing on a community needs survey with 4282 participants, we found a positive association between self-reported anxiety and seeking social support from health professionals, family and friends, and mediated sources. There was also a positive association between self-reported depression and seeking support from medical professionals and mediated sources but a negative association with seeking support from family and friends. Importantly, a positive indirect effect was observed between mental health and seeking support from family and friends through hesitancy, whereas negative indirect effects were documented between mental health and seeking support from health professionals and mediated sources through hesitancy. Theoretical, practical, and methodological implications were discussed.

## 1. Introduction

Although COVID-19 is a physical illness, it has consequential impacts on mental health [1,2,3]. The onset or aggravation of mental illnesses, such as depression or anxiety, during the pandemic is attributed to a variety of reasons, such as feelings of isolation due to restricted in-person interactions, trauma experienced or witnessed, family and economic stress, and reduced access to (mental) health care [3,4]. According to the World Health Organization [5], the pandemic triggered a quarter increase in the prevalence of depression and anxiety, with young people, women, and people with pre-existing health conditions at higher risk.

According to the buffering hypothesis [6], social support received from interpersonal and mediated sources can be protective against the negative effects of stress on mental health during the pandemic [4,7]. For instance, when suffering from stress, individuals tend to seek support from health professionals for expert insights [8,9] and from family and friends as trustworthy sources they can confide in [2]. Computer-mediated support groups are increasingly popular sources for social support, because of multiple advantages, such as overcoming geographic constraints, enabling text-based and asynchronous communication, maintaining anonymity, and allowing users to connect to others with similar characteristics and interests [10,11]. Seeking social support as a coping strategy was associated with lower levels of mental illnesses [2].

Despite the recognized benefits of social support on mental health, individuals grappling with depression and anxiety often encounter obstacles that hinder their access to support. A notable barrier is their reluctance to discuss mental health issues or seek assistance due to self-stigma and concerns about potential negative consequences impacting their self-image, personal relationships, or career prospects [12,13,14]. The concept of hesitancy, although pivotal in understanding social support-seeking behaviors, remains relatively understudied. Additionally, given the plethora of available support sources, understanding which sources individuals utilize when reporting depressive or anxiety symptoms and the role that hesitancy plays in source selection is essential, though currently unknown.

To address this gap in the literature, our study draws upon the channel complementarity theory (CCT) [15,16] to investigate how hesitancy mediates the associations between anxiety, depression, and the various social support systems individuals turn to during COVID-19. The significance of this research lies in its contribution to understanding the complex dynamics of support-seeking behaviors amid public health crises. This exploration not only extends the CCT within the social support literature but sheds light on mental health promotion. By unraveling the role of hesitancy as a mediating factor, our study provides valuable insights into designing targeted mental health-promotion campaigns or interventions to address barriers and encourage individuals, particularly from minority communities, to access crucial support during challenging times. In doing so, we can enhance mental health resilience and well-being in vulnerable populations, thereby fostering more inclusive and effective support systems during public health crises like the COVID-19 pandemic.

### 1.1. COVID-19 and Mental Health

The COVID-19 pandemic presented a variety of economic, social, and mental challenges for individuals’ physical and psychological well-being. Although a few studies examined the positive outcomes for well-being such as post-traumatic growth during the pandemic [17,18], the majority of extant research has reported the pandemic’s negative impacts on mental health. Individuals experienced psychological disequilibrium as a result of the disruption of their personal and professional goals and the uncertainty of goal achievement in light of the unpredictability of the pandemic [19,20]. In addition to the uncertainty, feelings of isolation, concern regarding the health threat, and overwhelming news exposure about COVID-19 all led to emotional stress and poor mental health [7,21].

A systematic review identified important gaps in the literature, including a dearth of communication studies on the mental health challenges that arose because of the pandemic [22]. Existing literature has primarily centered around explaining source selection when individuals seek pandemic-related information, but overlooked the exploration of barriers hindering individuals from selecting sources for social support in the context of COVID-19 [23]. This study aims to address this gap by investigating individuals’ hesitancy as a key barrier in seeking social support when experiencing anxiety or depression, thereby delving into how this hesitancy influences support seeking from interpersonal and mediated sources. Through this inquiry, our study extends research on CCT and social support by examining the sources individuals choose to receive social support from during the pandemic.

### 1.2. Social Support

Social networks exert a significant influence on an individual’s behavior [24]. The provision of support from social networks can influence people negatively, such as by prompting substance abuse [25], or positively, by encouraging actions such as seeking help for mental health [26]. Positive social support can strengthen individuals’ belief in being loved and cared for [27]. Individuals may seek informational, emotional, esteem-based, social network-based, and instrumental support [28], with an aggregate moderate effect size documented of the association between social support and individuals’ well-being [29].

The stress-buffering hypothesis posits that social support moderates the relationship between stress and health outcomes. In other words, “support ‘buffers’ (protects) individuals from the potentially pathogenic influence of stressful events (p. 310) [6]. Appropriate social support intervenes to protect individuals from the negative outcomes of stress by (a) helping a person reappraise a stressful event, mitigating the reaction to adversity at the start, and (b) alleviating the psychological and physiological responses of a stress reaction [6]. Because COVID-19 as a public health crisis represents a life event that individuals potentially appraised as threatening and subsequently experienced stress, empirical studies have supported the buffering hypothesis on pandemic-induced mental health issues. People who reported low individual- and neighborhood-level support had an increased likelihood of anxiety, depression, and post-traumatic stress during the pandemic [4], with seeking social support as a crucial coping mechanism for healthcare workers combating COVID-19-related psychological distress, trauma, anxiety, and depression [2]. Furthermore, social support was effective in buffering the impact of uncertainty on psychological disequilibrium [20], and acted as a protective factor for individuals’ mental health during the pandemic [30]. Because COVID-19 as a public health crisis represents a life event that individuals potentially appraised as threatening and subsequently experienced stress, the current research study explores social support through this lens. Since individuals have access to different sources of support, their preferences are important to understand, as discussed in the next section.

### 1.3. Channel Complementarity Theory (CCT) and Sources of Social Support

CCT, originally conceptualized to explain individuals’ source selection for obtaining health information, provides a rationale for source selection of social support. Individuals seek social support and acquire health information from three main sources: medical professionals, friends and families, and mediated sources (e.g., online support groups, social networking sites (SNSs)) [31]. Dutta-Bergman, who proposed the concept of channel complementarity, observed that in the aftermath of 9/11, individuals who exchanged social support with family and friends via telephone were also more likely to exchange support using the Internet [15]. In another study examining the impact of COVID-19 on mental health, Li et al. proposed that “individuals need to make full use of various social support resources to counteract the negative impact of the pandemic on mental health” (p. 11) [30]. According to CCT, individuals select sources of information that adequately meet their needs. Sources are assessed using four criteria: the ability to (1) offer access to medical expertise, (2) tailor the support to unique situations, (3) obtain support anonymously, and (4) access the source conveniently [16]. Research on COVID-19 and social support has explored three major sources of support and their impact on well-being.

#### 1.3.1. Support of Medical Professionals

Medical professionals, such as doctors, nurses, psychologists, and psychiatrists, are an important source of social support, particularly in a global pandemic where the stressful life event is directly related to physical health concerns and an overabundance of health information abounds. Individuals score medical professionals, including mental health specialists, high in all four categories of complementarity, with the two most salient categories being their credibility and ability to tailor expertise to patients’ unique circumstances [31,32]. People perceive medical professionals as credible, trustworthy sources of information, providing person-centered care, such as active listening, recognition of people’s experiences, and collaborative problem-solving, which facilitate patients’ perceptions of feeling supported [33]. However, despite people’s preference for interpersonal interactions with medical professionals [32,34], they were unable to access their healthcare providers in person or with ease during the pandemic [35], motivating them to seek support through other channels.

#### 1.3.2. Support of Friends and Family

For people of all ages, “family support is vital” (p. 11) [30]. Family and friends have long been considered significant interpersonal sources of support because people’s inability to name and discuss mental health symptoms is often considered a barrier to seeking professional help [36,37]. Additionally, friends and family are convenient to access and able to tailor advice and information to one’s unique stressor since they typically possess a large amount of personal knowledge about the individual [31]. When feeling being supported by family members, an individual is more likely to trust health information from them, which further motivates him/her to seek information from medical professionals and mediated sources [38]. Despite these advantages, support seekers usually feel a low level of anonymity during this process [31] and may be concerned about overburdening their family and friends [39], which contributes to their hesitancy to seek support.

#### 1.3.3. Support Through Mediated Sources

Although interpersonal social support can reduce burnout rates [2], increase self-esteem, lower depression rates [40], and help people manage and understand the symptoms of mental illness [41], when individuals practiced strict isolation during the pandemic, social connections took place through mediated platforms out of necessity. A recent trend analysis of the pandemic indicates a rise in online mental health support seeking from professionals, with a marked increase for those suffering from anxiety-related disorders [42]. When people were social distancing and isolating from their social networks, experts encouraged the use of SNSs to “enhance social support and connectedness” (p. 10) [43]. Research indicates that individuals turn to certain SNSs more than others to receive social support. For instance, Reddit users were more likely to seek social support than Facebook or YouTube subscribers during the COVID-19 pandemic [44]. A longitudinal study found that receiving social support online precipitated the tendency to seek online social support later [45]. Online support sources are favored because of their convenience, immediacy, and anonymity [31,32]. However, the majority of studies focus on one source of support, largely interpersonal [46,47], with a dearth of literature on the connections between multiple sources and CCT. The current research expands the literature by exploring the potential complementary use of all three sources for support during the pandemic and proposed H1 and H2 based on the buffering hypothesis [6].

U.S. adults’ self-reported depression (H1) and anxiety (H2) during the COVID-19 pandemic are positively associated with seeking social support from (a) health professionals, (b) family and friends, and (c) mediated sources.

### 1.4. Hesitancy to Seek Social Support

Although channel complementarity and social support research explore the selection of various sources and their connection to well-being, there is a paucity of scholarship that examines what factors serve as barriers to seeking support for mental health during public health crises and how they influence source selection. To fill this gap in the literature, this research project explores the role of hesitancy as a potential barrier. Hesitancy, observed among individuals dealing with mental health concerns, is conceptualized as a reluctance to seek help for mental health issues because of perceived negative outcomes [48]. Reasons that explain individuals’ hesitancy to seek support for mental health issues include but not limited to anticipated stigma from support providers or self-stigma, lack of mental health knowledge that leads to inability to express concerns, fear of negative consequences for their career or relationships, unwillingness to burden others, desire for autonomy and self-reliance, and a lack of access to medical professionals [12,13,14,36,37,49]. Thus, we proposed H3 as follows.

**H3:**  *U.S. adults’ self-reported (a) depression and (b) anxiety during the pandemic is positively associated with their hesitancy to seek social support.*

According to the health belief model (HBM) [50], hesitancy, as a barrier to support seeking, is negatively associated with individuals’ health behaviors, including seeking social support. Perceived barriers might be anything that stands in the way of the individual performing the action such as lack of financial resources, access to resources, or knowledge or expertise. The HBM purports that an individual decides to engage in particular health behaviors because they believe that they are susceptible to a health problem that carries severe consequences if no action is taken. Further, the perceived benefits of taking the proposed action (i.e., seeking social support) outweigh the costs involved with overcoming barriers to action, such as lack of financial resources, access to resources, or knowledge or expertise (Rosenstock et al., 1988). Of all the variables contained within the HBM, perceived barriers were identified in empirical research as the *strongest* predictor of (not performing) a certain health behavior [51,52]. Based on the HBM, H4 was proposed.

**H4:**  *U.S. adults’ hesitancy to seek social support is negatively associated with their seeking support from (a) health professionals, (b) family and friends, and (c) mediated sources.*

**Figure 1:**
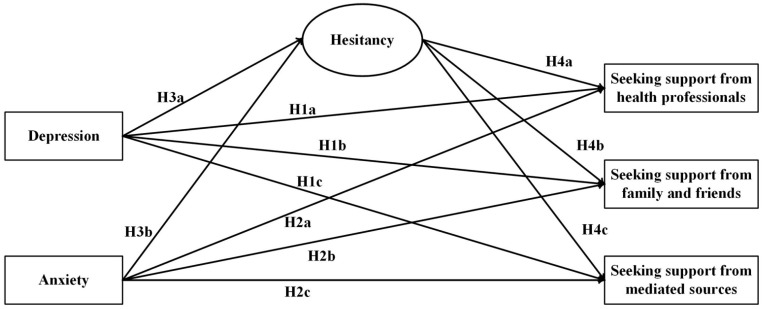
Hypothesized model-predicting sources of social support seeking. *Note*. Hesitancy = hesitancy to seek for social support. For visual clarity, participants’ age, gender, homelessness status, selected questionnaire version, and traumatic life event are included as control variables, but not presented in this figure.

Given the reasoning above, we will also test the following indirect effects (see Figure 1 for conceptual model).

U.S. adults’ hesitancy to seek social support mediates the associations between self-reported depression (H5) and anxiety (H6) and their seeking social support from (a) health professionals, (b) family and friends, and (c) mediated sources.

## 2. Methods

### 2.1. Procedure and Participants

The XXX County Department of Behavioral Wellness (blinded for review) in partnership with a broad array of community organizational providers and stakeholders including Cottage Health conducted a community needs survey in the fall of 2021. The community at large was invited to complete the survey, with no inclusion or exclusion criteria applied. Recruitment for the study employed a multifaceted approach encompassing diverse methods, aiming to maximize participant engagement and diversity within the survey, capitalizing on community connections, cultural understanding, and randomized selection processes through various communication channels. The multifaceted approach involves three steps. First, participants were invited through collaborating partner networks to leverage existing relationships. Second, promotoras were instrumental in facilitating recruitment at Spanish and Mixteco speaking community events, establishing cultural rapport. Finally, invitations were sent to randomly selected households via mail, text message, or phone call, ensuring equitable representation. To increase representation from Spanish-speaking and Mixteco communities, participants recruited through the use of promotoras received gift cards worth USD 25.

A total of 4282 respondents completed the survey (77.1% female). Age was measured as an ordinal variable including 1 = *6–11 years*, 2 = *12–17 years*, 3 = *18–29 years*, 4 = *30–49 years*, 5 = *50–69 years*, and *6 = 70 years or older* (*M* = 4.66, *SD* = 0.94; see Table 1 for details). The survey was made available in English and Spanish and was also conducted in the field by trusted outreach community survey providers for populations less likely to have access to an electronic survey. Field outreach providers were also able to translate the survey for Mixteco community members. In total, 502 participants (11.68% of total) completed the survey in Spanish and thus the survey language was controlled for in the analysis.

### 2.2. Measures

The data analyzed in this manuscript is derived from a larger survey assessing community health needs in XXX County, California (blinded for review). The variables of interest to this manuscript include socio-demographics (e.g., age, sex, homeless status), traumatic life events, mental health-related symptoms, hesitancy to seek support, and support-seeking behaviors in the last 12 months. Standardized measures to assess mental health were administered including the Generalized Anxiety Disorder (GAD-2) and the Patient Health Questionnaire (PHQ-2), which have been validated in diverse populations and across multiple languages, including Spanish.

#### 2.2.1. Depression

Depression was assessed using the two-item Patient Health Questionnaire-2 (PHQ-2) [53], a first-step approach to detecting depressive symptoms. Participants responded how often in the past two weeks they have felt “Little interest or pleasure in doing things” and “Down, depressed, or hopeless” on a 4-point scale (0 = No days; 3 = Nearly every day). Depression was measured by the sum of the two strongly correlated items (*r* = 0.76).

#### 2.2.2. Anxiety

The two-item Generalized Anxiety Disorder (GAD-2) scale was used to assess anxiety. The GAD-2, based on the GAD-7, is a brief initial screening tool for generalized anxiety disorder [54]. Participants responded how often in the past two weeks they have felt “Feeling nervous, anxious or on edge” and “Not being able to stop or control worrying” on a 4-point scale (0 = No days; 3 = Nearly every day). Anxiety was measured by the sum of the two strongly correlated items (*r* = 0.77).

#### 2.2.3. Hesitancy

Hesitancy to seek medical support was adapted from Kuhl et al. [55] and assessed through six items, such as “I think I should handle problems on my own”, and “Even if I had a problem, I would be too embarrassed or scared to talk about it” on a 1 (strongly disagree) to 4 (strongly agree) rating scale (Cronbach’s α = 0.82). Confirmatory factor analysis yielded good model fits (χ^2^ (7) = 45.02, *p* < 0.05; CFI = 0.97, TLI = 0.93, RMSEA = 0.051 (90% CI: 0.038, 0.066), SRMR = 0.023).

#### 2.2.4. Seeking Social Support

Participants were asked how often in the past 12 months they reached out to seven different social support systems to “help with emotional challenges or problems” on a 1 (0 times) to 4 (4 or more times) rating scale. An exploratory factor analysis suggested a two-factor solution. Specifically, seeking support from (a) a crisis hotline/text line and (b) a person or a group on social media were loaded on a different factor from the other five interpersonal sources, and labeled as the “mediated sources” factor (*M* = 1.17, *SD* = 0.45). Regarding the interpersonal sources, we further categorized (c) a friend, (d) a parent or relative, (e) a peer as the factor “family and friends” (*M* = 2.18, *SD* = 0.1.01; Cronbach’s α = 0.74) and (f) a medical doctor or nurse, and (g) a therapist, psychologist, or other mental health professional as the factor “medical professionals” (*M* = 2.22, *SD* = 1.09) based on the literature reviewed.

#### 2.2.5. Other Confounders

The structural equation model was adjusted for participants’ age, gender, and cultural orientation (assessed by participants’ selected questionnaire version (1 = Spanish; 0 = English) as a proxy),which were associated with social support seeking [56,57,58,59]. According to the stress-buffering hypothesis, homelessness status (1 = yes, 0 = no) and traumatic life events as stressors influencing social support-seeking behaviors [6,60,61] were also controlled for in the model. Traumatic life event (ACES) [62] was measured by the sum of four questions asking whether the participants have ever lived with anyone who (a) was mentally ill, (b) was alcoholic, (c) was sentenced to serve time in prison or other corrections facility, and (d) had separated or divorced parents (1 = yes, 0 = no).

### 2.3. Analysis

Structural equation modeling was employed to test H1–H4. Maximum likelihood estimation was applied with standard errors that were robust to non-normality. Model fit was examined using the following indices and criteria: values greater than 0.90 for the comparative fit index (CFI) and Tucker–Lewis index (TLI), and values smaller than 0.08 for the standardized root mean square residual (SRMR) and root mean square of approximation (RMSEA) [63,64,65].To test H5 and H6 investigating indirect effects, bootstrapping was implemented to obtain bias-corrected 95% confidence intervals for making statistical inferences about the mediation effects [66], which is better than other analytic approaches [67]. The number of replications was set to 5000 to ensure the precision of bias-corrected confidence intervals [67]. All analyses were performed using *Mplus* 8.0.

## 3. Results

### 3.1. Descriptive Statistics

Among the 4298 participants, 10.2% self-reported as homeless and 61.1% had at least one traumatic life event. On average, participants had a low level of depression, a moderate level of anxiety, but a moderate-to-high level of hesitancy to seek social support during the pandemic. Health professionals and family and friends are more frequent sources for seeking social support during the pandemic than mediated sources. Table 1 includes the descriptive statistics and Table 2 presents the zero-order correlation matrix of all continuous variables.

### 3.2. Hypothesis Testing

#### 3.2.1. Direct Effects

**Figure 2:**
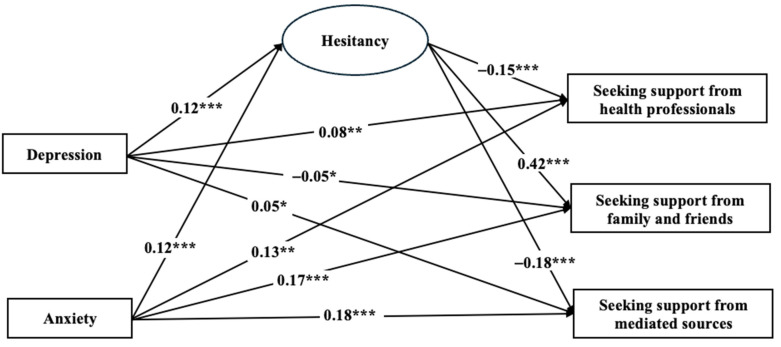
Standardized path coefficients of structural model-predicting sources of social support seeking. *Note*. Hesitancy = hesitancy to seek for social support. The structural model showed a good fit to the data: (*χ*^2^ (53) = 193.83, *p* < 0.001; CFI = 0.95, TLI = 0.91, RMSEA = 0.036 (90% CI: 0.031, 0.041), SRMR = 0.026). For visual clarity, participants’ age, gender, homelessness status, selected questionnaire version, and traumatic life event are included as control variables, but not presented in this figure. * *p* < 0.05, ** *p* < 0.01, *** *p* < 0.001.

Given that the hypothesized model fit the data very well (χ^2^ (53) = 193.83, *p* < 0.001; CFI = 0.95, TLI = 0.91, RMSEA = 0.036 (90% CI: 0.031, 0.041), SRMR = 0.026), we proceeded with examining the path coefficients to test the hypotheses. Participants’ self-reported depression was positively associated with seeking support from health professionals (*β* = 0.08, *p* < 0.01) and mediated sources (*β* = 0.05, *p* < 0.05) but negatively with seeking support from family and friends (*β* = −0.05, *p* < 0.05), supporting H1a and H1c, but not H1b. Consistent with H2a–c, participants’ self-reported anxiety was positively associated with seeking support from health professionals (*β* = 0.13, *p* < 0.001), family and friends (*β* = 0.17, *p* < 0.001), and mediated sources (*β* = 0.18, *p* < 0.001). The positive associations between participants’ hesitancy to seek social support and their depression (*β* = 0.12, *p* < 0.001) and anxiety (*β* = 0.12, *p* < 0.001) experienced during the pandemic respectively supported H3a and H3b. Interestingly, participants’ hesitancy to seek support was negatively associated with their support seeking from health professionals (*β* = −0.15, *p* < 0.001), and from mediated sources (*β* = −0.18, *p* < 0.001), but positively from family and friends (*β* = 0.42, *p* < 0.001). Therefore, H4a and H4c were supported while H4b was not. Figure 2 shows standardized path coefficients and statistical significance for individual paths in the hypothesized model.

#### 3.2.2. Indirect Effects

Results showed that participants’ hesitancy to seek social support significantly mediated the association between self-reported depression and seeking support from health professionals (ES = −0.018, 95%CIs (−0.030, −0.008)), family and friends (ES = 0.050, 95%CIs (0.026, 0.075)), and mediated sources (ES = −0.022, 95%CIs (−0.035, −0.011)), supporting H5. Similarly, H6 was supported given the significant indirect effects between anxiety and seeking support from health professionals (ES = −0.018, 95%CIs (−0.031, −0.008)), family and friends (ES = 0.051, 95%CIs (0.026, 0.077)), and mediated sources (ES = −0.022, 95%CIs (−0.036, −0.011)).

## 4. Discussion

Consistent with previous literature [12,13,14], our findings indicated that individuals’ experience of depression and anxiety was equally associated with higher hesitancy to reach out for support, which can be attributed to individuals’ self-stigma and concerns about the personal, interpersonal, and professional consequences of disclosing their mental health needs. The significant implications of COVID-19 for mental health [3,5] combined with the existing hesitancy of seeking support among individuals who struggle with mental health needs suggests a double-barreled public health crisis. Indeed, individuals who are in the most need of support are also those who are likely to be hesitant to seek the very support that could be beneficial for them.

Another interesting finding is that individuals reporting different mental health issues turned to different sources for support. The direct effects showed that people suffering from depression and anxiety are more likely to seek help from health professionals and mediated sources, showing consistency with the CCT [31], because health professionals and mediated support groups, high in all four complementarity characteristics—(a) access to medical expertise, (b) tailorability, (c) anonymity, and (d) convenience—are typical sources for support [33,45]. It is noteworthy that although family and friends remain an important support source for individuals reporting anxiety, depressed individuals are less likely to seek them out. This could be due to the increased stigma associated with depression relative to anxiety [8,13], the lack of anonymity of family and friends as helpers [31], and/or reluctance to burden and stress those beloved one for emotional charge [39], especially during such a challenging time. Generally speaking, individuals with self-reported anxiety held higher seeking support tendencies from all three major sources compared to those with self-reported depression. The discrepancy may be attributed to the lower anxiety stigma compared to the depression stigma [68], which could lay down more barriers for support-seeking behaviors among depressed individuals.

Through hesitancy to seek help, mental disorders (i.e., depression, anxiety) were negatively indirectly associated with seeking support from medical professionals and mediated sources but positively indirectly associated with seeking support from family and friends. The comparable effect sizes of the mediations from depression and anxiety respectively to support-seeking behaviors through hesitancy suggests similar mechanisms underlying these two mental disorders and social support seeking. These indirect effects may be explained by the unique attributes of family and friends as support sources, such as trustworthiness, non-judgmental attitude, genuine care, unconditional love, and sincere interest in support seekers’ well-being [39], which, however, are not taken into account by the extended CCT [16]. Such attributes may become particularly salient when support seekers experience barriers, such as hesitancy, to seek help due to self-stigma, concerns about the characteristics of support provider, lack of knowledge or training, and/or uncertainty about the next steps [36,37]. Although these factors can deter support seeking from health professionals and mediated sources, friends and family might be especially useful in these contexts. Our findings have important implications to the CCT, mental health campaigns or interventions and using community-based sample, which are detailed in the following section.

### 4.1. Theoretical, Practical, and Methodological Implications

Our study contributes to the CCT from two perspectives. First, although the CCT was proposed and has been primarily applied in the health information-seeking context [15,31], it could serve as a powerful tool to understand social support-seeking behaviors. After all, when seeking social support, the various sources are thought to serve different niches and present unique types of support, which is in line with the proposition of CCT [15]. The study is among the first that apply and extend the CCT to the social support context for a stigmatized topic. Besides the seminal study that proposed CCT in people’s use of traditional (e.g., telephone) and new(er) media (e.g., Internet) to satisfy their social support need following the 9/11 attacks [15], there is a paucity of research that examined social support as the communicative functions under the CCT framework. Considering that interpersonal and mediated sources could be better suited to handle specific needs [69], our investigation of the direct effects supported the CCT proposition that individuals who reported depression or anxiety turn to both sources for support as a “buffer” of their symptoms [6], which is the communicative functions that drive the selection of support sources in response to a crisis.

Second, despite Ruppel and Rains’ innovative extension of CCT by proposing and systematically testing the four complementarity characteristics (i.e., access to medical expertise, tailorability, convenience, anonymity) of health information sources [16,31], these four characteristics may not capture the full picture that predicts the complementary source use to serve a certain communicative function. Our direct effects indicated that individuals suffering from anxiety may turn to all three sources for support; however, when hesitancy to seek support is held, they seek support only from family and friends but less likely from professional or mediated sources. Such findings are at odds with the extensions, considering that both family/friends and mediated sources were rated as high in convenience by researchers [16], suggesting the likelihood of using both sources for a health purpose. Moreover, although family and friends were rated as low in all four traditional characteristics by health information seekers [31], they turned out to be the primary source for social support seekers with hesitancy due to stigmatized health issues. This finding suggests additional dimension(s) beyond the traditional four characteristics that explain or predict channel complementary use, such as trustworthiness, another key factor in addition to the access to medical expertise that affects a source’s credibility [70] or intimacy. Extending previous studies proposing characteristics that facilitate channel complementarity (explaining why using certain sources), the current study explored another factor (i.e., hesitancy) that explains why *not* using certain sources by innovatively examining this theory in the social support context for stigmatized health topics during a public health crisis.

The study also contributes to the social support literature in three folds. First, by identifying the mediating role of hesitancy in the association between mental disorders and support-seeking behaviors, the results highlighted the significance of hesitancy, which has been understudied [36]. Second, examining hesitancy as a barrier to seeking social support during the pandemic, our findings extended HBM [50] by specifying the distinct associations between perceived barriers and support seeking from different sources. Finally, despite the established effects of social support sources on mental health [30,71], there is a paucity of research examining the antecedents of source selection when seeking support. Therefore, our findings filled this gap by identifying an important cognitive predictor of sources sought for social support and elucidating the mechanisms between mental disorders and sources during the pandemic.

Several practical implications are also noteworthy. First, considering hesitancy mediating the association between mental disorders and support-seeking behaviors, it is important to incorporate hesitancy-reducing strategies in public health campaigns. Formative research based on in-depth interviews or focus groups would be beneficial to qualitatively pinpoint the specific reasons that drive the target population’s hesitancy [36,37] and ensure the campaign’s success. Second, the inconsistent direct and indirect effects between mental disorders and seeking social support emphasize the necessity to segment the audience at a deeper layer other than demographic characteristics [72]. Specifically, the target audience’s level of concern about seeking help should be considered an important parameter to segment the audience and select appropriate channels of social support when designing targeted campaigns or interventions. Finally, our findings emphasize the increased effort family and friends may need to make in reaching out to depressed individuals within their network given their unwillingness to seek support from close interpersonal contacts. Household-based health promotion partnered with community opinion leaders and delivered via easy-to-access channels, such as billboards or pamphlets, may potentially facilitate this process.

Methodologically, the use of a community-based sample is a strength of this study as it allows us to test the generalizability of the theoretical processes proposed. Indeed, many studies within the social sciences discipline and interpersonal communication more specifically have utilized samples that have restrictive diversity in terms of important demographic criteria [73]. Research utilizing community-based samples is not only useful for improving the validity of research methods but is essential for person-centered research in which there is equitable involvement of communities in health-related research [74].

### 4.2. Limitations and Future Research

Several limitations should be noted. First, the cross-sectional design limited the causal inferences between mental illness (i.e., depression, anxiety), hesitancy to seek social support, and selection of social support sources. Second, despite the innovative use of community needs surveys based on randomly selected households and the large sample size, our non-probability sample is limited to a specific geographic area (i.e., California) and therefore not nationally representative, which limits the generalizability of findings. Relatedly, cultural differences should be noted when examining help-seeking behaviors. For instance, while help seeking improved subjective well-being in independence-preferring countries (e.g., the U.S.), the help-seeking tendencies were found detrimental to well-being for people in interdependence-preferring countries, where preserving relational harmony and face concerns are the priorities [75]. Furthermore, the lockdown stringencies during the pandemic, which varied globally, could also impact individuals’ risk perceptions, anxiety, and depression [76], as well as their hesitancy and support-seeking behaviors, such that the barrier to obtaining social or medical support was significantly higher under more severe lockdown conditions [77]. Therefore, we encourage future studies to replicate our study in other states or countries or using nationally representative longitudinal panel data or experimental design. Third, our study only examined how social support behaviors are associated with two mental disorders (i.e., depression, anxiety) experienced during the pandemic, which is by no means a comprehensive investigation. Additionally, although we adjusted in the model for the major theory-driven individual characteristics that may influence the support-seeking behaviors, there may be other characteristic, such as self-control or resilient coping that may also influence individuals’ mental health status and support-seeking behaviors during the COVID-19 [76]. Researchers are encouraged to examine social support seeking for other mental disorders during a public health crisis, such as post-traumatic stress disorder [14] and explore other individual characteristics that may influence the mechanism under investigation. Fourth, both depression and anxiety were assessed by two-item scales, which although widely validated by previous studies [53,54,78,79], may not represent the complex symptoms and constrained us from analyzing them as latent constructs. Considering the analytic limitations of assessing exogenous (i.e., anxiety, depression) and endogenous variables (i.e., support seeking from health professionals, family and friends, and mediated sources respectively) as observed variables, further investigation could re-analyze them as latent constructs to correct measurement error. Finally, the direct and indirect relationships between mental disorders (i.e., depression, anxiety) and social support seeking were interpreted based on the existing literature and our best assumption. Future studies would benefit from qualitative investigation to better understand individuals’ preference for social support sources when they are or are not reluctant to seek support.

### 4.3. Conclusions

Overall, through the use of a community-based sample, this research was important in establishing the associations between mental health concerns, hesitancy to seek support, and actual reports of seeking support from different sources. Not only is it important to consider the different types of social support that individuals with mental health needs may seek but finding ways of reducing their hesitancy might be the key to cutting the Gordian knot of understanding why these individuals do not seek support.

## Figures and Tables

**Table 1:** Descriptive statistics of variables included in the structural equation models (N = 4298).

Variables	
Age (years; %)	
6–11	0.1
12–17	2.0
18–29	6.6
30–49	33.2
50–69	38.8
70+	19.3
Sex (%)	
Female	77.1
Male	22.7
Non-binary	0.6
Other	0.3
Questionnaire version (Spanish; %)	11.7
Homeless status (%)	10.2
Traumatic life event (ACES; *Min =* 0, *Max* = 4; *M* ± *SD*)	1.06 ± 1.07
Depression (PHQ-2; *Min =* 0, *Max* = 6; *M* ± *SD*)	1.49 ± 1.70
Anxiety (GAD-2; *Min =* 0, *Max* = 6; *M* ± *SD*)	1.96 ± 1.89
Hesitancy to seek social support (*Min =* 1, *Max* = 4; *M* ± *SD*)	2.99 ± 0.59
Seeking social support (*Min =* 1, *Max* = 4; *M* ± *SD*)	
From health professionals	2.22 ± 1.09
From family and friends	2.18 ± 1.01
From mediated sources	1.17 ± 0.45

*Note.* The percentage was calculated without including the missing values.

**Table 2:** Zero-order correlation matrix of all continuous variables.

Variable	α	1	2	3	4	5	6	7	8
Depression	--	--							
2. Anxiety	--	0.72 ***	--						
3. Hesitancy to seek for social support	0.82	0.22 ***	0.13 ***	--					
4. Seeking support from health professionals	--	0.26 ***	0.30 ***	−0.23 ***	--				
5. Seeking support from family and friends	0.74	0.16 ***	0.25***	−0.26 ***	0.57 ***	--			
6. Seeking support from mediated sources	--	0.18 ***	0.18 ***	−0.00	0.23 ***	0.26 ***	--		
7. Age	--	−0.10 ***	−0.19 ***	−0.09 ***	0.03	−0.12 ***	−0.13 ***	--	
8. Traumatic life event	--	0.22 ***	0.21 ***	−0.05 **	0.28 ***	0.24 ***	0.15 ***	−0.07 ***	--

*Note*. ** *p* < 0.01, *** *p* < 0.001. Age was measured as an ordinal variable in this study (1 = *6–11 years*, *6 = 70 years or* older). Cronbach’s α was not reported for variables that were measured by fewer than three items and was marked by “--” in that column.

## Data Availability

The data are available upon appropriate request.
